# Decursin, Identified via High‐Throughput Chemical Screening, Enhances Plant Disease Resistance via Two Independent Mechanisms

**DOI:** 10.1111/mpp.70101

**Published:** 2025-06-01

**Authors:** Yahui Ma, Yujie Zhao, Hanqi Huang, Yue Zhao, Rui Cao, Kunrong He, Lijuan Zhou, Yajin Ye

**Affiliations:** ^1^ State Key Laboratory of Tree Genetics and Breeding, Co‐Innovation Center for Sustainable Forestry in Southern China Nanjing Forestry University Nanjing China

**Keywords:** antipathogen, CERK1, chemical screening, decursin, small molecule

## Abstract

In order to overcome the damage caused by phytopathogens, plants have evolved a complex defence system to protect themselves, such as the two‐tiered innate immunity system. Chemical screening has led to the identification of plant immune‐priming compounds, which facilitate the functional dissection of the plant immune system and contribute to chemical control for plant diseases. In this study, we identified decursin, a coumarin natural product, through high‐throughput screening for activators of the expression of *FLG22‐INDUCED RECEPTOR KINASE 1* (*FRK1*). Decursin functions as a typical immune elicitor, triggering early immune responses, including a reactive oxygen species (ROS) burst, MAPK activation, and transcriptional reprogramming of defence genes. A targeted reverse genetic approach identified CERK1, a lysin motif receptor‐like kinase (LysM‐RLK), loss of function of which resulted in a significant reduction of decursin‐induced immune responses. Moreover, decursin was demonstrated to be ineffective in eliciting immune activation in the *lyk4 lyk5* mutant, a double mutant of two additional LysM‐RLKs. Molecular docking studies predicted that decursin may bind to CERK1 and LYK5. Decursin has been demonstrated to possess potent antiphytopathogenic properties, exhibiting pronounced growth inhibitory effects against several important plant fungal pathogens in vitro and in vivo, thereby protecting plants from damage caused by these pathogens. It can be concluded that decursin exerts its function through two independent mechanisms to enhance plant disease resistance, providing a potent agrochemical in disease control.

## Graphical abstract

Decursin operates through a dual mode of action: it simultaneously activates the plant immune system and directly inhibits fungal growth.

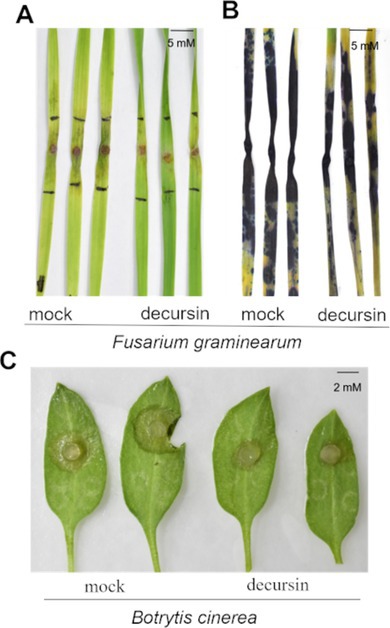

## 1 Introduction

In order to survive in the harsh ecosystem, plants have evolved a two‐tiered innate immunity system to combat invading harmful pathogens, and such immune system is mediated by two types of receptors: plasma membrane (PM)‐localised pattern‐recognition receptors (PRRs) and the intracellular nucleotide‐binding leucine‐rich repeat (NLR) resistome (Zhou and Zhang 2020). PRRs are capable of recognising conserved molecules on pathogens, which are known as pathogen‐/microbe‐, damage‐, and herbivore‐associated molecular patterns (PAMPs/MAMPs, DAMPs, and HAMPs, respectively). This recognition results in the activation of pattern‐triggered immunity (PTI). On the other hand, NLR receptors can activate effector‐triggered immunity (ETI) by recognition of effectors secreted by pathogens (Jones and Dangl 2006). Upon recognition by immune receptors, PAMPs and effectors typically elicit substantial downstream signalling cascades, including calcium influx, a surge of reactive oxygen species (ROS), mitogen‐activated protein kinases (MAPKs) activation, and reprogramming of immune responsive genes (Zhou and Zhang 2020).

A plethora of immune‐priming compounds have been identified through chemical screenings, thereby enhancing the comprehension of plant immune responses. A screening system based on *Pseudomonas syringae* pv. *tomato* DC3000 (Pto DC3000) *avrRpm1* identified several compounds that enhance effector‐triggered cell death, including salicylic acid, tiadinil, and nonsteroidal anti‐inflammatory drugs (Noutoshi et al. 2012; Ishihama et al. 2021). Sulfamethoxazole was identified by chemical screenings for small molecules that protect *Arabidopsis* from infection by Pto DC3000 using *Arabidopsis* seedlings (Schreiber et al. 2008). In addition to phenotype‐based screenings, defence‐responsive gene‐based screenings have identified several compounds that activate defence responses. 3,5‐Dichlorobenzoic acid and 2‐(5‐bromo‐2‐hydroxy‐phenyl)‐thiazolidine‐4‐carboxylic acid, two immune‐priming chemicals identified through *CaBP22*::*GUS*‐based screening, have been shown to trigger disease resistance against pathogens (Knoth et al. 2009; Bektas et al. 2016). The small molecule [5‐(3,4‐dichlorophenyl) furan‐2‐yl]‐piperidine‐1‐ylmethanethione (DFPM), identified via a chemical screen for the inhibition of abscisic acid (ABA)‐responsive gene expression, activates plant immunity by disrupting ABA signal transduction at the Ca^2+^ signalling level (Kim et al. 2011). Concurrently, direct binding‐based screening has also been conducted to identify chemicals that can effectively induce plant immunity. Maya1 and Maya2, two activators of FLAGELLIN‐SENSING 2 (FLS2), were identified through screening small molecules that could bind to FLS2 (Lee et al. 2024). Using yeast two‐hybrid‐based chemical screening, the small molecule zaractin was identified, which has been shown to activate the ZAR1‐mediated ETI pathway in plants through its ability to enhance the interaction between ZRK3/PBL27 (Seto et al. 2021).

However, the majority of the identified plant immune‐priming compounds that have been previously identified are synthetic chemicals. The use of these chemicals in crops may result in the presence of residues in plants, which may cause environmental concerns. In this study, we employed an immune‐marker‐gene‐based chemical biology approach to identify novel bioactive small molecules that activate plant immunity. The screening of a collection of natural and synthetic chemicals yielded several candidate immune inducers. Decursin, a natural product derived from angelica plants (Chu et al. 2024) was identified as a particularly potent activator of plant immunity. In a manner similar to DAMP, decursin elicits a transient ROS burst, MAPK activation, and the expression of immune‐responsive genes. By integrating targeted reverse genetics and biochemical techniques, our findings revealed that AtCERK1 and AtLYK4/LYK5 were the plasma membrane proteins responsible for decursin perception. Moreover, decursin functions as a novel antimicrobial chemical, inhibiting the growth of several destructive phytopathogens, including *Fusarium graminearum*, *Botrytis cinerea*, and *Fusarium oxysporum*. In conclusion, our findings demonstrate that decursin exerts protective effects on plants against pathogen invasion through two independent mechanisms, thereby offering a promising agrichemical for the control of plant diseases.

## 2 Results

### 2.1 Decursin Was Identified to Activate *FRK1* Expression Through High‐Throughput Screening

**FIGURE 1:**
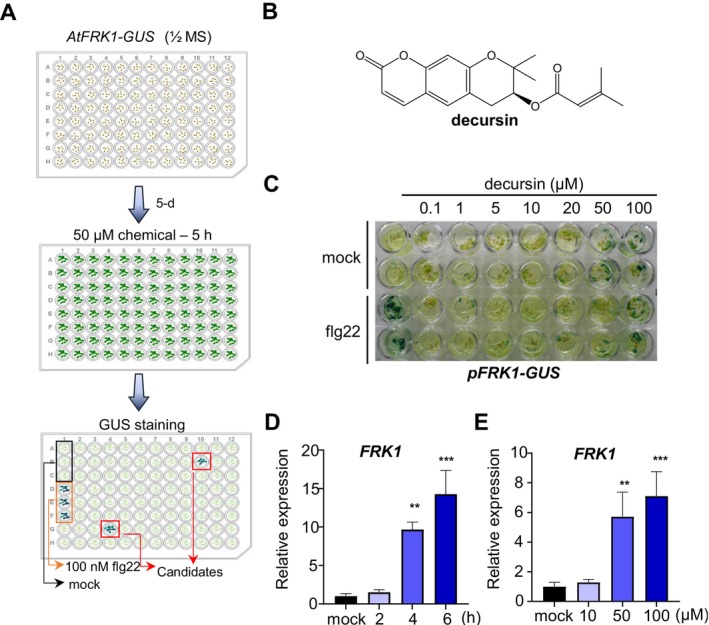
Secondary metabolite decursin activates *FRK1* expression. (A) The flowchart of the screening for the inducers of *pFRK1‐GUS*. Individual chemical was added to each well with the 5‐day‐old *pFRK1‐GUS* seedlings and incubated for 5 h. Afterwards, β‐glucuronidase (GUS) staining was conducted. (B) The chemical structure of decursin. (C) Decursin activates *pFRK1‐GUS* expression. After 5 days' growth in 1/2 × MS medium, transgenic *pFRK1‐GUS* seedlings were soaked with different concentrations of decursin as indicated. After 4‐h chemical treatments, GUS staining was conducted to detect the expression of *FRK1*. 100 nM flg22 was used as positive control and dimethyl sulphoxide (DMSO) was used as solvent control. (D) Decursin induces *FRK1* expression in a dose‐dependent manner. Col‐0 seedlings were soaked with the indicated concentrations of decursin 4 h prior to examining the transcript levels of *FRK1* using reverse transcription‐quantitative PCR. Values are means ± SEM, *n* = 3 (biological replicates), **p < 0.01, ***p < 0.001 (two‐tailed Student's *t* test). (E) Decursin activates *FRK1* expression in a time‐dependent manner. Col‐0 seedlings were soaked with 50 μM decursin at 0, 2, 4 and 6 h prior to examining the transcript levels of *FRK1*. Values are means ± SEM, *n* = 3 (biological replicates), **p < 0.01, ***p < 0.001 (two‐tailed Student's *t* test).

To identify small molecules that activate plant immunity, we developed a chemical screening system that relied on the inducible expression of the innate immunity marker gene *FLG22‐INDUCED RECEPTOR‐LIKE KINASE 1* (*FRK1*) (Bjornson et al. 2021). For each well of a 96‐well plate, 100 μL of 1/2 × Murashige and Skoog (MS) medium was added, and approximately 10 *pFRK1‐GUS* seeds were sown in each well. After a 5‐day growth period, 50 μM designated chemical from the commercial chemical libraries was added to each well and incubated for 5 h before β‐glucuronidase (GUS) staining. A positive control of 100 nM flg22 was used (Figure 1A). Chemical libraries of approximately 10,000 compounds were screened using the *pFRK1‐GUS* seedlings, which harbour a reporter construct consisting of the *FRK1* promoter fused to *GUS*. The most potent inducers of *pFRK1‐GUS* were selected for further validation in a second‐round screening process. Following these screenings and validations, we identified 10 chemicals capable of inducing *pFRK1‐GUS* expression. In this study, we reported one of these chemicals, decursin, which effectively activated *pFRK1‐GUS* expression, exhibiting a dose‐dependent response (Figure 1B,C). The induction of *FRK1* expression was confirmed by reverse transcription‐quantitative PCR (RT‐qPCR) (Figure 1D). Furthermore, the induction of *FRK1* expression by decursin was in a time‐dependent manner (Figure 1E).

Decursin is a pyranocoumarin compound, which is primarily extracted from the roots of the genus *Angelica*, including the Chinese and Korean traditional medicinal herb *Angelica decursiva* and *Angelica gigas* (Hata and Sano 1969; Ahn et al. 1996; Shehzad et al. 2018). The biosynthesis pathway of decursin commences with phenylalanine ammonia‐lyase (PAL), which catalyses the conversion of phenylalanine to cinnamic acid (Park et al. 2012). Decursin and its analogue decursinol angelate (DA) are the products of their precursor decursinol (Figure S1). Despite their structural similarity with decursin, neither decursinol nor DA was observed to induce *pFRK1‐GUS* expression (Figures S2A,B and S3A,B).

### 2.2 Decursin Induces Early Immune Responses in *Arabidopsis*

**FIGURE 2:**
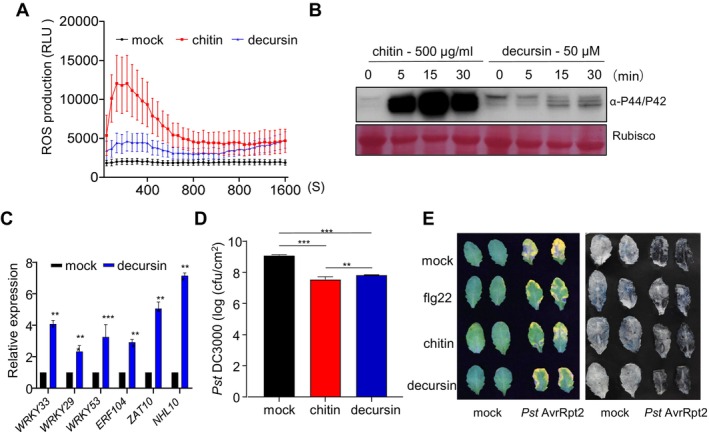
Decursin functions as an elicitor, triggering immune responses. (A) Reactive oxygen species (ROS) production was measured from Col‐0 leaf disc for 30 min after treatment with 50 μM decursin. Data are mean ± SE (*n* = 8). 500 μg/mL chitin was used as positive control. (B) Decursin induces MAPK activation. *Arabidopsis* seedings were soaked with 50 μM decursin for 0, 5, 15 and 30 min prior to anti‐p44/42 immunoblot. 500 μg/mL chitin was used as positive control. The protein bands stained with Ponceau S were used as a loading control. (C) Decursin induces the expressions of defence‐related genes in *Arabidopsis thaliana* . The expressions of indicated genes were quantified using reverse transcription‐quantitative PCR in the wild‐type with or without 50 μM decursin treatment. *AtEF1a* was used an internal control. Data are mean ± SE (*n* = 3). ***p < 0.001 (two‐tailed Student's *t* test). (D) Decursin enhances plant resistance to *Pseudomonas syringae* pv. *tomato* (Pst) DC3000, leaves of Col‐0 plants were infiltrated with mock or 50 μM decursin 2 days prior to the injection of Pst DC3000 inoculation. Experiments were performed three times with similar results. Bacterial populations were determined 2 days after inoculation. Data are mean ± SE (*n* = 3). ***p < 0.001 (two‐tailed Student's *t* test). (E) Decursin enhances plant resistance to Pst AvrRpt2. 4‐week‐old Col‐0 leaves were infiltrated with indicated chemicals. After 24 h chemical treatments, Pst AvrRpt2 (OD_600_ = 0.02) was infiltrated into the chemical pretreated leaves, and hypersensitive response (HR) phenotypes were detected at 24 h post‐inoculation. Cell death was detected using trypan blue staining.

Next, we examined whether decursin could elicit other hallmarks of the plant immune response. The production of ROS in leaf discs treated with decursin was quantified. The results demonstrated that decursin induced a weak ROS burst in *Arabidopsis* leaf discs, and the ROS burst‐curve showed a weaker level compared with that elicited by chitin (Figure 2A). The activation of MAPKs was examined using western blotting, which demonstrated that the phosphorylation of MAPKs occurred following the application of 50 μM decursin to *Arabidopsis* seedlings (Figure 2B). The expression of six PTI marker genes (*WRKY33*, *WRKY29*, *WRKY53*, *ERF104*, *NHL10*, and *ZAT10*) was evaluated through RT‐qPCR. The results demonstrated that the expression of all these genes was significantly induced following decursin treatment in comparison with mock‐treated seedlings (Figure 2C), indicating that decursin could induce the transcriptional reprogramming of defence‐related genes in a manner similar to other elicitors (Bjornson et al. 2021). The impact of decursinol and DA on these early immune responses was also investigated. With the exception of a weak activation of MAPK phosphorylation by decursinol, no other elicitations were observed, including the ROS burst and the *FRK1* expression (Figures S2A–E and S3A–D). This indicates that the specific immune‐inducing activity of decursin is not present in its analogues.

To further determine the potential involvement of decursin in plant immunity, an examination was conducted to test whether decursin influenced the growth of the virulent strain Pst DC3000. The pre‐infiltration of decursin 2 days prior to bacterial infection was observed to enhance the resistance of *Arabidopsis* against Pst DC3000, thereby reinforcing the role of decursin as a plant immune‐priming compound in plants (Figure 2E). In addition, plants were primed with 50 μM decursin for 24 h, after which the ETI‐inducing strain Pst AvrRpt2 was infiltrated into the plants. In accordance with the previous report, the hypersensitive response (HR) elicited by Pst AvrRpt2 was markedly reduced by the priming of fig22 and chitin (Wang et al. 2023). Decursin exhibited a comparable effect to that of flg22 or chitin (Figure 2F). The attenuation of cell death, as indicated by trypan blue staining, was evident following the priming of decursin, flg22, or chitin, which was consistent with the reduced HR phenotype (Figure 2F). In conclusion, these findings suggest that decursin, a secondary metabolite derived from the genus *Angelica*, functions as a novel natural product in activating plant immune responses.

### 2.3 LysM Receptor Kinases AtCERK1 and AtLYK4/LYK5 Are Essential for the Decursin Responses

**FIGURE 3:**
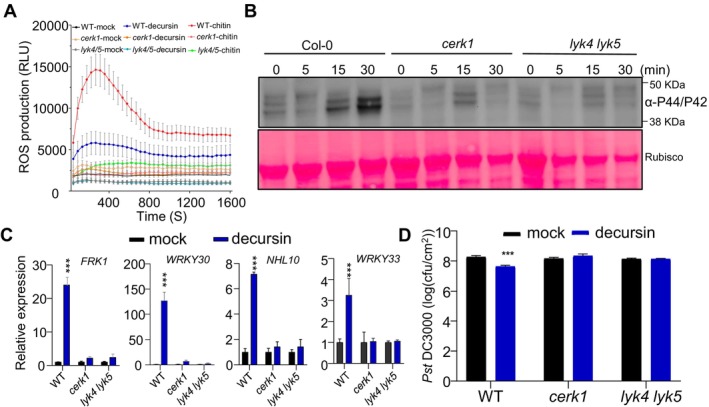
*Atcerk1* and *Atlyk4 lyk5* mutant plants are defective in decursin‐triggered immune responses. (A) The production of reactive oxygen species (ROS) was quantified in leaf discs of Col‐0, *Atlyk4 lyk5*, and *Atcerk1* mutant plants for 30 min following the treatment of 50 μM decursin. 0.5% dimethyl sulphoxide (DMSO) was used as mock control. The data are presented as the mean ± SE (*n* = 8 biological replicates). (B) The *Atlyk4 lyk5* and *Atcerk1* mutant plant displays a reduction in MPK3 and MPK6 phosphorylation in response to 50 μM decursin compared to the wild‐type plant over a time course from 0 to 30 min. The phosphorylation of MPK3 and MPK6 was detected using an antibody that recognises phospho‐p44/p42 MAPK. Ponceau S staining (bottom panel) was employed as a protein loading control. The experiment was conducted three times, yielding comparable results each time. (C) The relative expression of *FRK1*, *WRKY30*, *NHL10* and *WRKY33* in the wild‐type (WT), *Atcerk1* and *Atlyk4 lyk5* mutant plants with and without 50 μM decursin treatment. *AtEF1α* was used as the internal control. The data are presented as mean ± SE (*n* = 3 biological replicates). (D) Leaves from plants of indicated genotype were infiltrated with mock control or 50 μM decursin 2 days prior to the *Pseudomonas syringae* pv. *tomato* (Pst) DC3000 inoculation. Bacterial populations were determined 2 days after inoculation. Four times experiments were performed with similar results. Data are mean ± SE (*n* = 4 biological replicates). ***p < 0.001 (two‐tailed Student's *t* test).

Given the observation that the immune responses induced by decursin are similar to those responses elicited by PAMPs/DAMPs, it can be hypothesised that certain PRRs may mediate the activity of decursin. To test this hypothesis, a targeted reverse genetics approach was employed to identify PRR mutants that were unable to respond to decursin. A panel of eight PRRs with known patterns was selected for the analysis, including FLAGELLIN SENSING 2 (FLS2), LRR‐RK EF‐TU RECEPTOR (EFR), LRR‐RKs PEP1‐RECEPTOR (PEPR1) and PEPR2, RECEPTOR‐LIKE PROTEIN 23 (RLP23), CHITIN ELICITOR RECEPTOR KINASE 1 (CERK1), LIPOOLIGOSACCHARIDE‐SPECIFIC REDUCED ELICITATION (LORE), DOES NOT RESPOND TO NUCLEOTIDES 1 (DORN1), and HYDROGEN PEROXIDE INDUCED CA^2+^ INCREASE 1 (HPCA1) (Gómez‐Gómez and Boller 2000; Yamaguchi et al. 2006; Zipfel et al. 2006; Miya et al. 2007; Wan et al. 2008; Choi et al. 2014; Kutschera et al. 2019; Wu et al. 2020). We employed decursin‐induced MAPK phosphorylation as a marker to assess the responsiveness of these PRR mutants to decursin. Consequently, we observed that decursin was unable to induce MAPK phosphorylation in *cerk1* mutants, in contrast to the wild type (WT) (Figure 3B). Following this, other immune responses were further tested in the *cerk1* mutant following decursin treatment. Similarly, decursin was observed to lose its capacity to induce ROS production in the *cerk1* mutant (Figure 3A).

Moreover, the LysM‐PRR LYK5 has been identified as a primary receptor for chitin perception (Wan et al. 2012; Cao et al. 2014; Xue et al. 2019; Yang et al. 2022). Accordingly, we investigated the potential involvement of LYK5 and its homologue LYK4 in the perception of decursin. As with the *cerk1* mutants, plants in which AtLYK4 and AtLYK5 had been mutated exhibited compromised responses to decursin, including a decrease in ROS production and MAPK phosphorylation (Figure 3A,B). These findings are consistent with the observation that the *cerk1* and *lyk4 lyk5* mutant plants exhibited a compromised response with regard to decursin‐induced *AtFRK1*, *AtWRKY30*, *AtWRKY33*, and *AtNHL10* expression compared to the WT plants (Figure 3C). Pst DC3000 was inoculated into WT, *cerk1*, and *lyk4 lyk5* mutant plants with or without prior administration of decursin. The *cerk1* and *lyk4 lyk5* mutants exhibited resistance to Pst DC3000 at levels comparable to those of the WT (Figure 3D). However, only the WT plants exhibited enhanced resistance to bacterial infection when they were pretreated with decursin, thereby confirming that AtCERK1, AtLYK4/AtLYK5 are involved in decursin signalling (Figure 3D).

### 2.4 Molecular Docking Between Decursin and AtLYK5/AtCERK1

**FIGURE 4:**
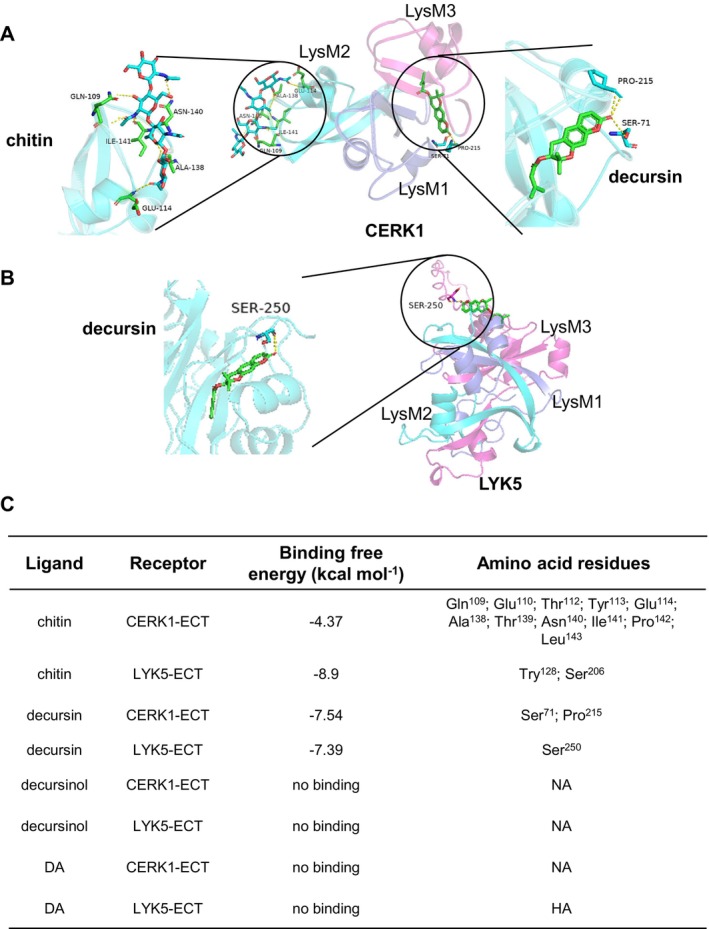
Molecular docking between decursin and CERK1/LYK5. (A) Schematic representation of the molecular docking of decursin with CERK1, with chitin serving as a control. Chitin is predicted to dock into the LysM2 domain, whereas decursin is predicted to interact with the LysM1 and LysM3 domains. The amino acids involved in these interactions are highlighted. (B) The interaction between decursin and LYK5, stimulated by docking. Serine^250^ forms a hydrogen bond with decursin. (C) The binding free energy and interacting amino acids between the indicated ligands and receptors. DA, decursinol angelate. NA indicates that the data is not available.

In *Arabidopsis*, chitin is perceived by the CERK1/LYK5 cell membrane receptor complex through direct binding to them, with chitin binding to LYK5 with a higher affinity than to CERK1 (Liu et al. 2012; Cao et al. 2014). To explore whether decursin might similarly interact with CERK1/LYK5, we performed computational docking simulations. The crystal structure of the CERK1 ectodomain was employed to predict direct binding (Liu et al. 2012). Given the absence of structural data for LYK5, we employed the AlphaFold protein structure prediction tool (Jumper et al. 2021) to generate a model of the LYK5 ectodomain. The docking simulation indicates that decursin could form a stable complex with both CERK1 and LYK5, with binding energies of −7.54 kcal mol^−1^ and −7.39 kcal mol^−1^, respectively (Figure 4A–C). Decursin was predicted to dock into the shallow surface groove between LysM1 and LysM3 of CERK1, in which two residues, Ser‐71 and Pro‐215, were predicted to form hydrogen bonds with decursin (Figure 4A). However, this interaction appears less extensive than that of chitin, which engages 11 residues based on crystallographic data (Liu et al. 2012). In addition, decursin was modelled to interact with LYK5‐LysM3, possibly forming a hydrogen bond with Ser‐250 (Figure 4B). As a control, neither decursinol nor DA demonstrated the capacity to bind to CERK1 or LYK5, which is consistent with the specific immune‐inducing activity of decursin, but not its two analogues (Figure 4C). These findings indicate that decursin may bind to LYK5 and CERK1, thereby transducing immune signalling.

### 2.5 Decursin Shows Strong Antifungal Activity and Triggers ROS Production in Fungal Cells

**FIGURE 5:**
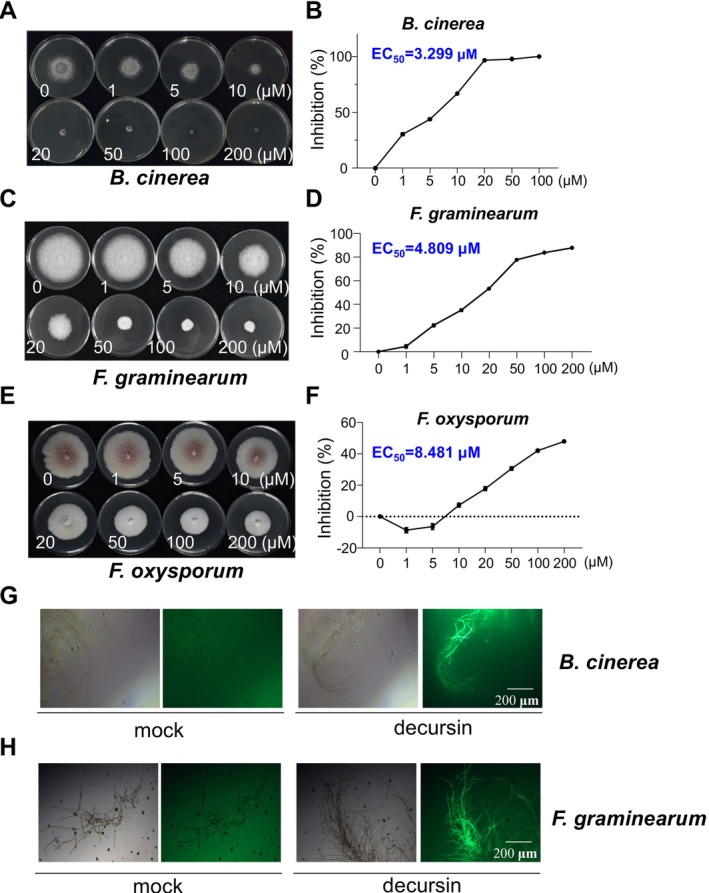
Decursin inhibits mycelial growth of phytopathogenic fungi in vitro. The effect of decursin on the growth of fungi was observed on media with varying concentrations of the compound: (A) *Botrytis cinerea*, (C) *Fusarium graminearum*, and (E) *Fusarium oxysporum*. Fresh fungal mycelia in agar plugs with a diameter of 3 mm were inoculated onto the medium with the indicated concentration of decursin. The inhibition curves of *B. cinerea* (B), *F. graminearum* (D), and *F. oxysporum* (F) under different concentrations of decursin are presented. The half maximal effective concentration (EC_50_) for each fungus was calculated and is indicated in blue. Decursin induces reactive oxygen species (ROS) production in *B. cinerea* (G) and *F. graminearum* (H). The detection of ROS was facilitated through dichlorodihydrofluorescein diacetate (DCFH‐DA) staining. The detection of green fluorescent protein (GFP) signals was conducted following a 2‐h treatment with 10 μM decursin on the mycelia.

**FIGURE 6:**
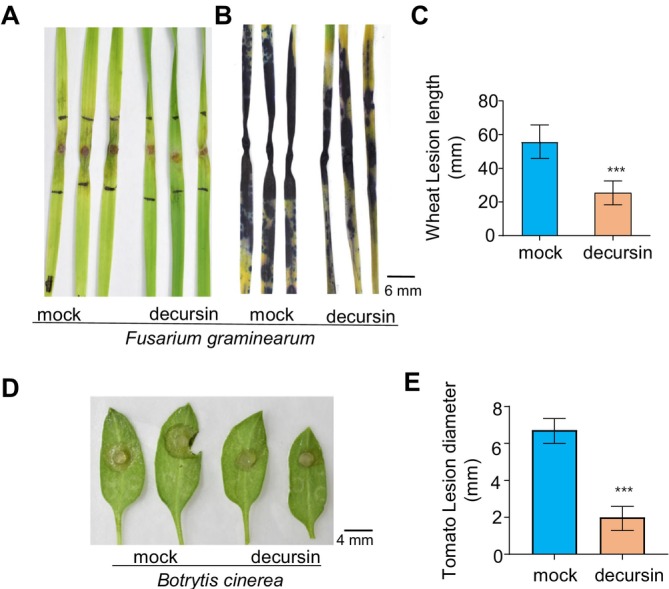
Decursin protects plants from phytopathogen invasion. (A) Wheat leaves were infiltrated with 1% dimethyl sulphoxide (DMSO) or 100 μM decursin 24 h prior to the inoculation of 3 mm agar plugs of fresh *Fusarium graminearum* mycelia. The appearance of disease symptoms was photographed at 5 days post‐inoculation (dpi). (B) Trypan blue staining of the wheat leaves shown in (A). (C) The quantification of lesion length of wheat leaves in (A). The data are presented as the mean ± SE (*n* = 3 biological replicates), with a two‐tailed Student's *t* test applied. (D) Decursin enhances tomato resistance to *Botrytis cinerea*. Prior to the inoculation of 3 mm *B. cinerea* fresh mycelial blocks, tomato leaves were infiltrated with either 1% DMSO or 100 μM decursin, 24 h in advance. Photographic documentation of disease symptoms was conducted 24 h post‐inoculation. (E) The lesion diameter of (D). The data are presented as mean ± SE (*n* = 4 biological replicates), with a two‐tailed Student's *t* test.

Several coumarins have been identified as phytoanticipins or phytoalexins, and these antimicrobial coumarins have been discovered to play a significant role in the plant's chemical defence strategy (Stringlis et al. 2019). To investigate the antimicrobial activity of decursin, an in vitro study was conducted to assess the effects of decursin on the growth of a variety of different plant‐pathogenic fungal and bacterial species. Eight fungal pathogens were evaluated, including *Botrytis cinerea*, *Fusarium graminearum*, and *Fusarium oxysporum* (Table 1). Six of the pathogenic fungi exhibited a pronounced inhibition in growth when treated with decursin, as evidenced by the suppression of germ tube elongation (Figure 5A–F; Figure S4). The necrotrophic plant pathogen *B. cinerea* was the most sensitive fungus towards decursin, as evidenced by the lowest EC_50_ (Table 1). It is noteworthy that decursin did not exhibit inhibitory activity against the pathogenic bacteria tested, including Pst DC3000 and *Ralstonia solanacearum* (Figure S5). This suggests that decursin may have a selective effect, targeting fungal growth while leaving bacterial proliferation uninhibited. Furthermore, the activity of decursinol, the hypothetical precursor of decursin, and DA, another downstream product of decursinol, was tested. At a dose of 50 μM, which demonstrated strong inhibitory activity for decursin, decursinol did not affect the growth of *F. graminearum* or *B. cinerea* (Figure 6A–E; Figure S6). Similarly, DA was observed to inhibit the mycelial growth of both *F. graminearum* and *B. cinerea* , indicating that the downstream products of decursinol, but not decursinol itself, possess antimicrobial activity (Figure S6). With regard to the phytopathogens under examination, the efficacy of decursin was found to be considerably higher than that of scopoletin, a well‐known phytoalexin (Sun et al. 2014). Indeed, 50 μM of scopoletin was observed to have a negligible impact on the growth of these fungal organisms (Figure S7).

**TABLE 1:** The effects of decursin on the growth of different phytopathogens.

Pathogen	Inhibition regression equation	*R* ^2^	EC_50_ (μM)	EC_95_ (μM)
*Botrytis cinerea*	*y* = 17.416*x* − 7.4639	0.93	3.299	5.883
*Fusarium graminearum*	*y* = 14.242*x* − 18.493	0.97	4.809	7.970
*Fusarium oxysporum*	*y* = 8.4584*x* − 21.738	0.90	8.481	13.801
*Glomerella cingulata*	*y* = 11.017*x* − 19.043	0.97	6.267	10.352
*Sclerotinia sclerotiorum*	*y* = 9.7926*x* − 22.517	0.82	7.405	12.001
*Valsa mali*	*y* = 7.2041*x* − 16.702	0.80	9.259	15.505
*Alternaria solani*	NA	NA	NA	NA
*Colletotrichum higginsanum*	NA	NA	NA	NA
*Pseudomonas syringae* pv. *tomato* DC3000	NA	NA	NA	NA
*Ralstonia solanacearum*	NA	NA	NA	NA

Mounting evidence indicates that ROS‐mediated oxidative damage plays a pivotal role in the antifungal activity of plant bioactive compounds. This assertion is evidenced by the ability of ROS to inflict irreversible oxidative damage to vital components and macromolecules found in plants, such as lipids, proteins and DNA (Kohanski et al. 2008; Chen et al. 2023; Mittler et al. 2022; Yang, Wang, and Liu 2022; Yang, Yang, et al. 2022). Consequently, we detected the production of ROS in *B. cinerea* and *F. graminearum* following decursin treatment. In the absence of decursin, no ROS generation was detected in either *B. cinerea* or *F. graminearum*, whereas ROS production was detected in the mycelia following a 2‐h incubation of decursin, which may be a critical factor contributing to the antifungal activity of decursin (Figures 5G and 6H).

### 2.6 Decursin Protects Crops From Phytopathogen Invasion

To further investigate the in vivo plant protection activity of decursin, tests were conducted on wheat and tomato. The administration of decursin prior to the inoculation of *F. graminearum* resulted in the mitigation of disease symptoms and a reduction in cell death in wheat leaves, as evidenced by the staining of cells with trypan blue (Figure 6A–C). Similarly, the pretreatment of decursin was observed to provide protection for tomato leaves against infection by necrotrophic *B. cinerea* (Figure 6D,E).

Together, our findings suggest that decursin's protective effect in plants against phytopathogens is achieved through two distinct mechanisms: the activation of plant immunity and the inhibition of fungal growth. Consequently, the combination of these two distinct mechanisms contributes to the overall protection of plants against phytopathogens following decursin treatment.

## 3 Discussion

In the present study, decursin was identified through chemical screening using the *pFRK1‐GUS* reporter system. Decursin was observed to elicit rapid ROS production, rapid and transient MAPK activation, and the expression of immune marker genes in *Arabidopsis*, indicating that it activates PTI (Figures 1 and 2). A targeted reverse genetic approach was employed to identify the CERK1‐LYK4/5 module as a sensor complex for decursin, as decursin was observed to lose its eliciting activity in the *cerk1* and *lyk4 lyk5* mutants. In addition to its immune activation activity, decursin was identified as a novel antimicrobial chemical, exhibiting robust antifungal activity against a range of significant phytopathogens. Molecular docking suggests a possible interaction between decursin and the CERK1/LYK5 receptors. However, further experimental validation is necessary to confirm the possible binding. In addition, the detailed molecular mechanisms and signalling cascade remain largely unexplored. Understanding these downstream events is crucial for elucidating the complete picture of how decursin enhances plant immunity. A number of coumarins have been demonstrated to act as phytoalexins or phytoanticipins, thereby affording protection to plants from pathogen invasion. In vitro studies have demonstrated that decursin is capable of suppressing a number of phytopathogenic organisms at relatively low concentrations. In comparison to other coumarin‐type antimicrobial compounds, such as phytoalexin scopoletin, decursin has been demonstrated to exhibit a higher degree of phytopathogen inhibitory activity in vitro against the phytopathogens that were tested. This suggests that decursin may be a more potent agrochemical.

Thus, our findings suggest a synergistic two‐layered defence mechanism: (1) direct antifungal action through disruption of fungal cell wall integrity and hyphal growth (Figure 5); (2) immune priming via CERK1/LYK5‐mediated signalling (Figure 4). We propose that the antifungal activity reduces initial pathogen pressure, allowing more efficient resource allocation for immune responses, whereas immune activation reinforces antifungal defences through cell wall fortification and antimicrobial production. This dual functionality makes decursin particularly promising for sustainable agriculture, as it combines immediate pathogen suppression with long‐term resilience through immune priming, potentially reducing reliance on conventional fungicides. With increasing attention to environmental protection and sustainable agricultural development, the development and use of more environmentally friendly and safer agrochemicals have become essential trends. Decursin, as a natural product, may have the advantages of good biodegradability and minimal environmental residues, aligning with the requirements of sustainable agriculture. Its potential application in agriculture may not only reduce the use of chemical pesticides and lower environmental pollution but also improve the quality and safety of agricultural products, promoting sustainable agricultural development.

In soil and water, decursin may be metabolised by microorganisms into harmless by‐products. However, we have not yet conducted detailed studies on the specific degradation pathways and rates of decursin. Future research will include assessments of decursin's degradation kinetics under various environmental conditions. In addition, we primarily focused on decursin's inhibitory effects on plant pathogens and its immune‐activating effects on plants. However, a comprehensive evaluation of decursin's potential impacts on non‐target organisms, including beneficial microbes, is necessary. Preliminary in vitro experiments indicated that decursin significantly inhibits the growth of several plant‐pathogenic fungi but does not affect the growth of bacteria, such as Pst DC3000 and *R. solanacearum* . This suggests that decursin may have some selectivity, primarily targeting fungal pathogens. Nevertheless, we need to conduct more extensive ecological toxicity assessments across a broader range of biological communities to ensure minimal impact on ecosystems.

Given that the concentration of decursin in the roots of angelica plants is considerably higher than the EC_50_ values (Park et al. 2012; Rhee et al. 2010), it can be inferred that in the angelica plants decursin is in a physiological dose for the protection of the plant. However, further studies should be conducted to determine the activity of decursin in the plants that produce it, such as *A. decursiva*. The present study primarily focused on the model plant, *Arabidopsis thaliana* , which may not fully represent the complex immune responses in crop plants. Nevertheless, the efficacy of decursin in enhancing disease resistance in major crops, such as wheat and tomato has been demonstrated. However, further research is necessary to ascertain the impact of decursin on a broader range of agricultural species, which would enhance our understanding of decursin.

## 4 Experimental Procedures

### 4.1 Plant Materials and Growth Conditions

All *Arabidopsis* plants used in this study are in the Columbia (Col‐0) genetic background. Seeds of *cerk1* and *lyk4 lyk5* mutants were described as before (Wan et al. 2008; Cao et al. 2014). The *bbc* and *fec* seeds were described as before (Xin et al. 2016). *Arabidopsis* seeds were surface‐sterilised with 30% NaOCl for 15 min, washed five times with sterile water, and stratified for 2 days before growing in medium (1/2 × MS salts, 0.5% sucrose, pH 5.7) at 22°C with a 16‐h photoperiod in a growth chamber or greenhouse. Wheat ( *Triticum aestivum* ‘Jimai‐22’) and tomato ( *Solanum lycopersicum* ‘Kuwait’) were used in this study. Wheat seedlings were cultured in a greenhouse at 20°C–25°C with a photoperiod of 16 h light/8 h dark.

### 4.2 Chemicals

The chemical libraries used in this study were purchased from TargetMol (catalogue no. L6010) and Selleck (catalogue no. L1400; L3600). Decursin, purchased from Shanghai Yuanye Bio‐Technology (CAS 5928‐25‐6), was dissolved in dimethyl sulphoxide (DMSO). Decursinol, purchased from RENI Pharmaceutical Technology Co. Ltd. (CAS number: 23458‐02‐8), was dissolved in DMSO. DA, purchased from Shanghai Yuanye Bio‐Technology (CAS 130848‐06‐5), was dissolved in DMSO. The flg22 peptide, with a purity of 95.3%, was synthesised by Genescript and dissolved in deionised water. Chitin (Sigma‐Aldrich) was dissolved in deionised water.

### 4.3 GUS Staining

The putative *FRK1* promoter sequence (*proPRK1*; c. 2000 bp upstream of starting codon ATG) was amplified from Col‐0 genomic DNA using gene‐specific primers (Table S1). The *proFRK1:GUS* construct was cloned into the pGWB3 vector and introduced into Col‐0 plants using the floral dip method. Transgenes showed strong flg22‐induced expression and were propagated. The homozygous T_3_ seedlings were used for screening. The histochemical detection of GUS activity was performed as previously described (Ye et al. 2020).

### 4.4 Total RNA Extraction and RT‐qPCR

Total RNA was extracted from seedlings (more than 60 seedlings for each sample) treated with chemicals using the Jiangsu Cowin Biotech Ultrapure RNA Kit. RNA was reverse transcribed and quantified using Vazyme HisScript II Q RT SuperMix for qPCR (+gDNA wiper); 1 μg DNase‐treated RNA was reverse transcribed with PrimeScript reverse transcriptase (TaKaRa). A 1 μL aliquot of the cDNA solution along with the ChamQ SYBR qPCR Master Mix Quantification Kit (TaKaRa) and the CFX Connect Real‐Time PCR Detection System were used to conduct the RT‐qPCR analysis according to the manufacturer's instructions. Changes in the target gene transcript levels were determined using the 2^−ΔΔ*C*t^ method, with the previously studied *EF1a* gene as a reference control (Ye et al. 2020). The primers used for RT‐qPCR are shown in Table S2.

### 4.5 ROS Burst Assay

To measure ROS burst, 4‐week‐old *Arabidopsis* leaf discs with an area of 0.125 cm^2^ were incubated overnight under low light in 96‐well plates containing 50 μL deionised water. The deionised water was then replaced with a 100 μL reaction solution consisting of 20 μM L‐012 (TargetMol Chemicals Inc.), 10 μg/mL horseradish peroxidase (Beyotime Biotechnology, P2369), and either flg22, chitin, or chemicals. ROS production was subsequently measured using a High Sensitivity Plate Luminescence Detector (BLT Lux‐P110). Each data point represents the results from 40 leaf discs in a biological replicate.

### 4.6 MAPK Kinase Activation Assay

Seven‐day‐old *Arabidopsis* seedlings or 4‐week‐old leaf discs with an area of 0.125 cm^2^ were treated with the chemicals or DMSO (as a control). The samples were collected at different time points by quick freezing in liquid nitrogen and stored at −80°C. After grinding the samples into a fine powder using zirconia beads (3 mm diameter) in a FastPrep 24 sample grinder, protein extraction was performed using a buffer containing 50 mM Tris–HCl pH 7.5, 150 mM NaCl, 10% glycerol, 2 mM EDTA, 5 mM dithiothreitol (DTT), 1 × EDTA‐free complete protease inhibitor cocktail (Roche), and 1% Triton X‐100. Western blot analysis was conducted by loading equal amounts of protein onto a 4%–20% SDS‐PAGE gel. Phosphorylated MPK3 and MPK6 proteins were detected using a rabbit monoclonal anti‐Phospho‐p44/42 antibody (Cell Signalling technology; 1:2000). Goat anti‐rabbit IgG horseradish peroxidase (HRP) (Abmart, 1:12,000) was employed as the secondary antibody. Protein images were captured using the Tanon 3600 imaging system. All experiments were repeated three times with consistent results.

### 4.7 Bacterial Infection Assessment

Pst DC3000 was inoculated onto King's B plates (King's B medium 12.5 g/L; agar 15 g/L; glycerol 10 mL/L) and incubated for 48 h at 28°C in an incubator. Single colonies were selected and inoculated in 4 mL of KB liquid medium. The inoculated cultures were then placed in a shaker at 200 rpm and 28°C for 12–16 h. Once the OD_600_ reached 0.6–0.8, the cells were centrifuged and diluted in 10 mM MgCl_2_ buffer. The bacteria with an OD_600_ value of 0.002 were gently injected into the fully developed 4‐week‐old *Arabidopsis* leaves using a 1 mL needle‐free syringe. The bacterial solution on the surface of the *Arabidopsis* was absorbed using filter paper, after which the plants were returned to the growth chamber. Following a 3‐day period, the infected *Arabidopsis* leaves were harvested and the leaf disks measuring 0.6 cm in diameter were excised using a hole punch. The ground tissues were diluted a specified number of times with 10 mM MgCl_2_ and plated on KB plates. These plates were then placed in an incubator at 28°C, and the number of colonies on each plate was counted after 48 h. For each independent experiment, a minimum of eight plants were tested per data point. The experiments were conducted in triplicate.

### 4.8 Molecular Docking Analysis

The three‐dimensional structure of decursin as a ligand molecule was obtained from PubChem. The crystal structure of the CERK1 protein was obtained from the PDB database and designated as the receptor molecule. Semiflexible docking of ligand molecules and receptor molecules was conducted using the molecular docking software AutoDock v. 4.2.6, with consideration of the active pocket site. A visual representation of the compound‐target binding model was generated using PyMol v. 3.0.4.

### 4.9 Fungicidal Activity

Fresh 3 mm mycelial blocks of each phytopathogen were cultured on potato dextrose agar (PDA) plates with varying concentrations of decursin. Following a 72‐h incubation period at 28°C, the mycelial growth of the cells was evaluated in accordance with the methodology outlined in previous studies (Zhou et al. 2020).

### 4.10 Assays for the Assessment of Phytopathogenic Diseases in Wheat and Tomato

Prior to fungal inoculation, the tomato plants were infiltrated with either 1% DMSO or 100 μM decursin. Fresh mycelial blocks of *B. cinerea* (2 mm) were inoculated onto the leaves of 4‐week‐old tomato plants. The mycelial blocks were positioned at the centre of the adaxial surface of a true leaf. Twenty‐four hours after inoculation, the lesion areas were quantified using ImageJ software. Infection assays with *F. graminearum* were conducted using wheat plants cultivated at 25°C. Fresh mycelial blocks (diameter of 3 mm) were inoculated onto the leaves of 4‐week‐old wheat plants. Following inoculation, the leaves were placed in high‐humidity trays. At 5 days post‐inoculation (dpi), lesion diameter on the leaves was quantified using ImageJ software.

### 4.11 In Vivo ROS Imaging With L‐012

To quantify the production of ROS, 7‐day‐old *Arabidopsis* seedlings were incubated for 24 h under low light conditions in 12‐well plates containing 1 mL deionised water. Subsequently, the deionised water was replaced with 100 μL reaction solution comprising 20 μM L‐012 (TargetMol Chemicals Inc., T22096), 10 μg/mL HRP (Beyotime Biotechnology), and either flg22, chitin, or chemicals. Subsequently, ROS production was quantified using the Tanon 3600 imaging system. The data presented represent the results obtained from eight to 10 seedlings in a single biological replicate.

## Conflicts of Interest

The authors declare no conflicts of interest.

## Supporting information


**Figure S1.** Proposed biosynthetic pathway for decursin. PAL, phenylalanine ammonia‐lyase; C4H, cinnamate 4‐hydroxylase.

**Figure S2.** The effects of decursinol on plant immune responses. (A) The chemical structure of decursinol. (B) Decursinol did not activate *pFRK1‐GUS* expression. *pFRK1‐GUS* transgenic seedlings were treated with varying concentrations of decursinol for 5 h prior to the detection of the β‐glucuronidase (GUS) signal. (C) Decursinol did not induce the expression of *FRK1* and *WRKY30*. Col‐0 seedlings were soaked with the indicated concentrations of decursinol for 4 h prior to the quantification of the transcript levels of *FRK1* and *WRKY30* using reverse transcription‐quantitative PCR (RT‐qPCR). (D) Images of reactive oxygen species (ROS) burst in wild‐type (WT) seedlings elicited with decursin and decursinol. (E) The mutations of *cerk1* and *lyk4 lyk5* did not reduce MPK3 and MPK6 phosphorylation in response to 50 μM of decursinol. The phosphorylation of MPK3 and MPK6 was detected using an antibody that recognises phospho‐p44/p42. Ponceau S staining (bottom panel) was employed as a protein loading control. The experiment was conducted three times, yielding comparable results each time.

**Figure S3.** The effects of decursinol angelate on plant immune activation. (A) The chemical structure of decursinol angelate. (B) Decursinol angelate did not activate *pFRK1‐GUS e*xpression. *pFRK1‐GUS* transgenic seedlings were treated with varying concentrations of decursinol angelate for 5 h prior to the detection of the β‐glucuronidase (GUS) signal. (C) Reactive oxygen species (ROS) production was measured from Col‐0 leaf disc for 30 min after treatment with 50 and 100 μM decursinol angelate. Data are mean ± SE (*n* = 8). 100 nM flg22 was used as positive control. (D) Decursinol angelate did not induce the activation of MAPKs. *Arabidopsis* seedings were soaked in 50 μM decursinol angelate for 0, 5, 15 and 30 min prior to anti‐p44/42 immunoblot analysis. Ponceau S staining was employed as a protein loading control (bottom panel).

**Figure S4.** The sensitivity of phytopathogens to decursin. Mycelial growth of *Glomerella cingulata* (A), *Sclerotinia sclerotiorum* (C) and *Valsa mali* (E) treated with different concentrations of decursin. (B) Growth curve of *G. cingulata* in (A). (D) Growth curve of *S. sclerotiorum* in (C). (F) Growth curve of *V. mali* in (E). The half maximal effective concentration (EC_50_) for each fungus was calculated and is indicated in blue. (G,H) *Alternaria solani* and *Colletotrichum higginsanum* are not sensitive to decursin. For all these in vitro growth assays, 3 mm‐diameter mycelial discs were inoculated onto the medium with the indicated concentration of decursin.

**Figure S5.** The growth of *Pseudomonas syringae* pv. *tomato* (Pst) DC3000 and *Ralstonia solanacearum* under decursin treatment. (A) Single colony of Pst DC3000 was inoculated on the media with different concentrations of decursin. (B) The growth curve of Pst DC3000 with indicated concentration of decursin. Data are mean ± SE (*n* = 3). (C) The growth of single colony of *R. solanacearum* treated with different concentrations of decursin. (D) The growth of *R. solanacearum* under the indicated concentration of decursin in a liquid medium. Data are mean ± SE (*n* = 3).

**Figure S6.** The effects of decursin analogues on the growth of *Botrytis cinerea* and *Fusarium graminearum* in vitro. Fresh fungal mycelia with a diameter of 3 mm were inoculated onto the medium with the indicated concentration of the analogues of decursin.

**Figure S7.** The growth of phytopathogens treated with scopoletin. (A) Chemical structure of scopoletin. (B) The growth of *Valsa mali*, *Sclerotinia sclerotiorum* and *Botrytis cinerea* on the medium with 50 μM scopoletin. Discs of 3 mm diameter of fungal mycelia were inoculated on the medium.

**Table S1.** List of primers for plasmid construction.

**Table S2.** Primers used in the reverse transcription‐quantitative PCR.

## Data Availability

The data that support the findings of this study are available from the corresponding author upon reasonable request.
