# Correction to Decursin, Identified via High‐Throughput Chemical Screening, Enhances Plant Disease Resistance via Two Independent Mechanisms

**DOI:** 10.1111/mpp.70165

**Published:** 2025-10-30

Ma, Y., Zhao, Y., Huang, H., Zhao, Y., Cao, R., He, K., Zhou, L., and Ye, Y. “Decursin, Identified via High‐Throughput Chemical Screening, Enhances Plant Disease Resistance via Two Independent Mechanisms.” *Molecular Plant Pathology* 2025; 26: e70101.

In the ‘Acknowledgements’ section, the funding grant number to L. J. Zhou from Natural Science Foundation of Jiangsu Province should be BK20220418, not BK2022045268.

Here is the correct ‘Acknowledgements’ section:

We thank Dr. Yangrong Cao (Huazhong Agricultural University) for providing *lyk4 lyk5* double mutant; Dr. Gang Li (Nanjing Agricultural University) for providing *Fusarium graminearum* strain; and Dr. Zhiqin Liu (Fujian Agriculture and Forestry University) for providing *Ralstonia solanacearum* strain. This work is funded by National Natural Science Foundation of China (32300249 to L.J. Zhou and 32200213 to Y.J. Ye) and Natural Science Foundation of Jiangsu Province (BK20220418 to L.J. Zhou and BK20220417 to Y.J. Ye).

We apologise for this error.

