# Supplementary material for: Decursin, Identified via High‐Throughput Chemical Screening, Enhances Plant Disease Resistance via Two Independent Mechanisms
Source: Mol Plant Pathol. 2025 Jun 1;26(6):e70101. doi: 10.1111/mpp.70101 (PMC12127108; doi:10.1111/mpp.70101)
Supplement: Supplementary file 1 — Figure S1. Proposed biosynthetic pathway for decursin. PAL, phenylalanine ammonia‐lyase; C4H, cinnamate 4‐hydroxylase. [file MPP-26-e70101-s002.pdf]

Supplementary Figure 1

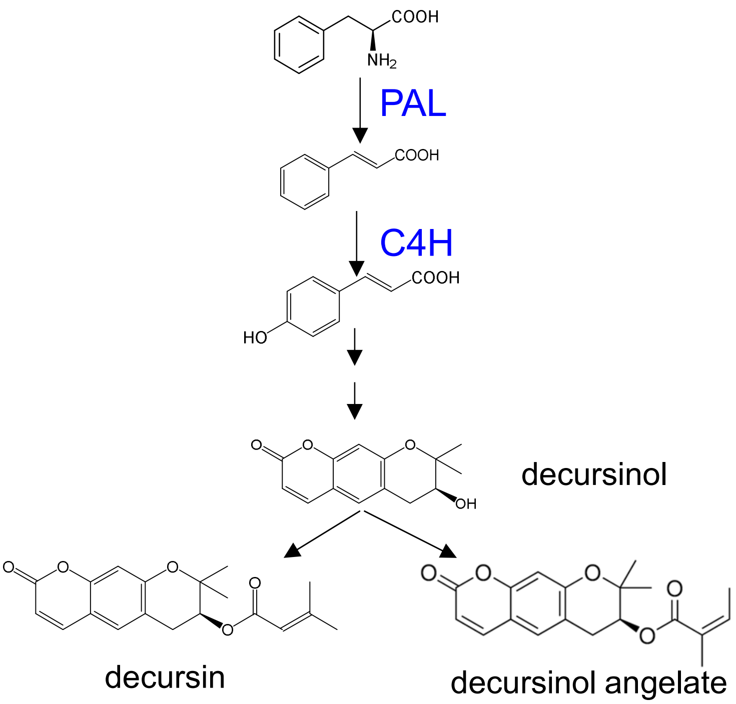

Supplementary Figure 1. Proposed biosynthetic pathway for decursin. PAL phenylalanine ammonia-lyase; C4H cinnamate 4-hydroxylase.
