# Supplementary material for: Decursin, Identified via High‐Throughput Chemical Screening, Enhances Plant Disease Resistance via Two Independent Mechanisms
Source: Mol Plant Pathol. 2025 Jun 1;26(6):e70101. doi: 10.1111/mpp.70101 (PMC12127108; doi:10.1111/mpp.70101)
Supplement: Supplementary file 2 — Figure S2. The effects of decursinol on plant immune responses. (A) The chemical structure of decursinol. (B) Decursinol did not activate pFRK1‐GUS expression. pFRK1‐GUS transgenic seedlings were treated with varying concentrations of decursinol for 5 h prior to the detection of the β‐glucuronidase (GUS) signal. (C) Decursinol did not induce the expression of FRK1 and WRKY30. Col‐0 seedlings were soaked with the indicated concentrations of decursinol for 4 h prior to the quantification of the transcript levels of FRK1 and WRKY30 using reverse transcription‐quantitative PCR (RT‐qPCR). (D) Images of reactive oxygen species (ROS) burst in wild‐type (WT) seedlings elicited with decursin and decursinol. (E) The mutations of cerk1 and lyk4 lyk5 did not reduce MPK3 and MPK6 phosphorylation in response to 50 μM of decursinol. The phosphorylation of MPK3 and MPK6 was detected using an antibody that recognises phospho‐p44/p42. Ponceau S staining (bottom panel) was employed as a protein loading control. The experiment was conducted three times, yielding comparable results each time. [file MPP-26-e70101-s003.pdf]

Supplementary Figure 2

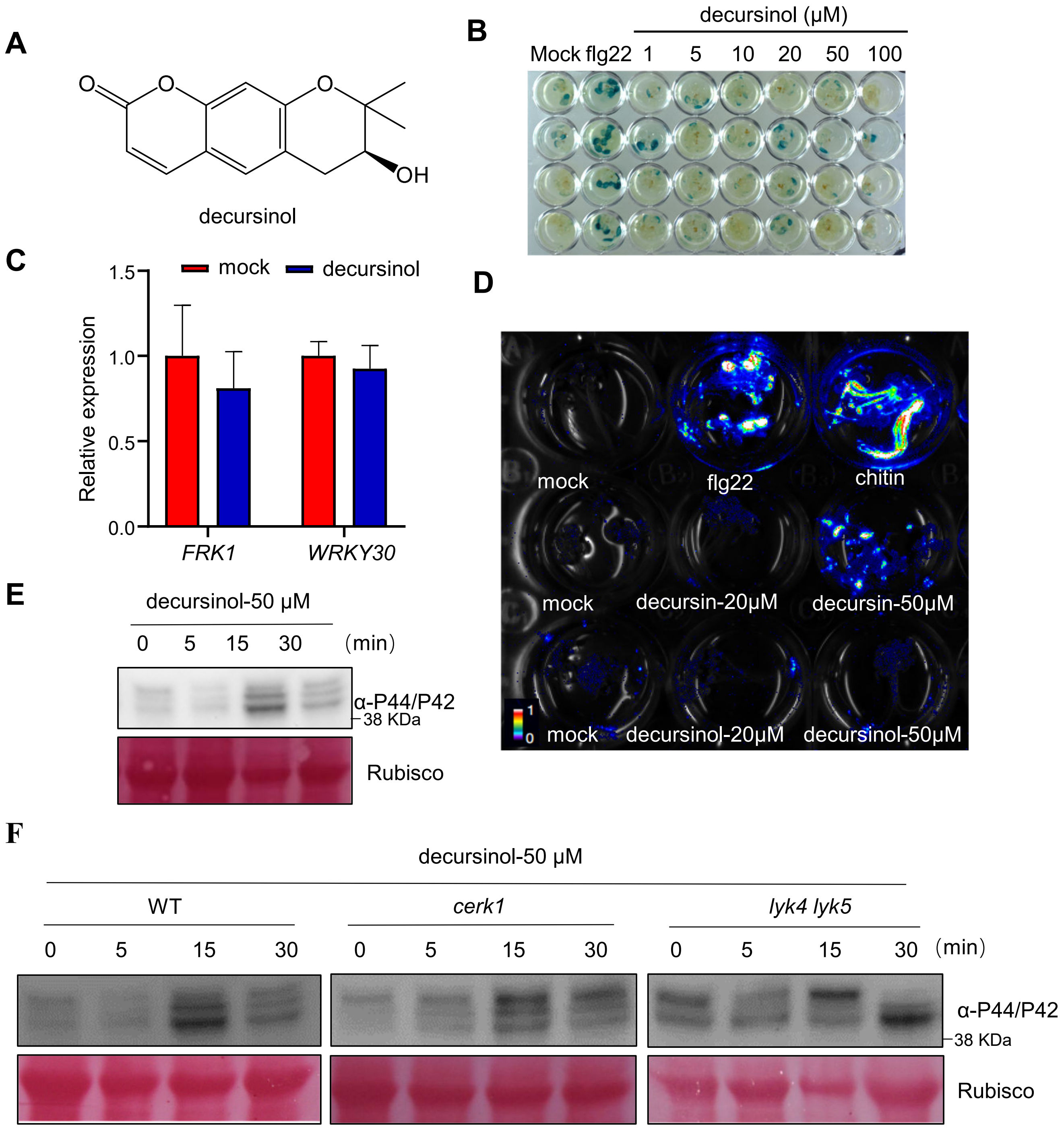

Supplementary Figure 2. The effects of decursinol on plant immune responses.

(A) The chemical structure of Decursinol. (B) Decursinol did not activate *pFRK1-GUS* expression. *pFRK1-GUS* transgenic seedlings were treated with varying concentrations of decursinol for a period of 5 hours prior to the detection of the GUS signal. (C) Decursinol did not induce the expression of *FRK1* and *WRKY30*. Col-0 seedlings were soaked with the indicated concentrations of decursinol for a period of 4 hours prior to the quantification of the transcript levels of *FRK1* and *WRKY30* using qRT-PCR. (D) Images of ROS burst in wild-type (WT) seedlings elicited with decursin and decursinol. (E) The mutations of *cerk1* and *lyk4 lyk5* did not reduce MPK3 and MPK6 phosphorylation in response to 50 μM of decursinol. The phosphorylation of MPK3 and MPK6 was detected using an antibody that recognizes phospho-p44/p42. The Ponceau S staining (bottom panel) was employed as a protein loading control. The experiment was conducted three times, yielding comparable results each time.
