# Supplementary material for: Decursin, Identified via High‐Throughput Chemical Screening, Enhances Plant Disease Resistance via Two Independent Mechanisms
Source: Mol Plant Pathol. 2025 Jun 1;26(6):e70101. doi: 10.1111/mpp.70101 (PMC12127108; doi:10.1111/mpp.70101)
Supplement: Supplementary file 3 — Figure S3. The effects of decursinol angelate on plant immune activation. (A) The chemical structure of decursinol angelate. (B) Decursinol angelate did not activate pFRK1‐GUS expression. pFRK1‐GUS transgenic seedlings were treated with varying concentrations of decursinol angelate for 5 h prior to the detection of the β‐glucuronidase (GUS) signal. (C) Reactive oxygen species (ROS) production was measured from Col‐0 leaf disc for 30 min after treatment with 50 and 100 μM decursinol angelate. Data are mean ± SE (n = 8). 100 nM flg22 was used as positive control. (D) Decursinol angelate did not induce the activation of MAPKs. Arabidopsis seedings were soaked in 50 μM decursinol angelate for 0, 5, 15 and 30 min prior to anti‐p44/42 immunoblot analysis. Ponceau S staining was employed as a protein loading control (bottom panel). [file MPP-26-e70101-s008.pdf]

Supplementary Figure3

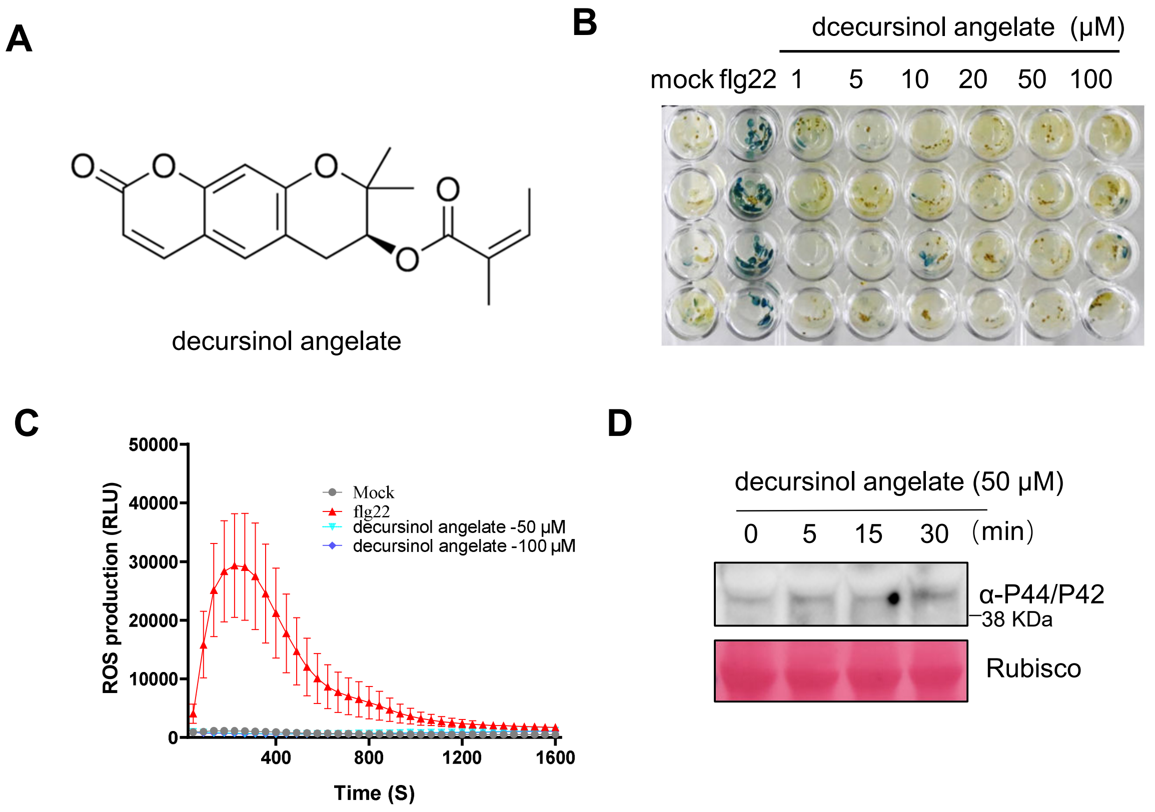

Supplementary Figure 3. The effects of decursinol angelate on plant immune activation.

(A) The chemical structure of decursinol angelate. (B) Decursinol angelate did not activate *pFRK1-GUS* *e*xpression. *pFRK1-GUS* transgenic seedlings were treated with varying concentrations of decursinol angelate for a period of 5 hours prior to the detection of the GUS signal. (C) ROS production was measured from Col-0 leaf disc for 30 min after treatment with 50 and 100 μM decursinol angelate. Data are mean ± SE (n = 8). 100 nM flg22 was used as positive control. (D) Decursinol angelate did not induce the activation of MAPKs. Arabidopsis seedings were soaked in 50 μM decursinol angelate for 0, 5, 15 and 30 minutes prior to anti-p44/42 immunoblot analysis. The Ponceau S staining method was employed as a protein loading control (bottom panel).
