# Supplementary material for: Decursin, Identified via High‐Throughput Chemical Screening, Enhances Plant Disease Resistance via Two Independent Mechanisms
Source: Mol Plant Pathol. 2025 Jun 1;26(6):e70101. doi: 10.1111/mpp.70101 (PMC12127108; doi:10.1111/mpp.70101)
Supplement: Supplementary file 4 — Figure S4. The sensitivity of phytopathogens to decursin. Mycelial growth of Glomerella cingulata (A), Sclerotinia sclerotiorum (C) and Valsa mali (E) treated with different concentrations of decursin. (B) Growth curve of G. cingulata in (A). (D) Growth curve of S. sclerotiorum in (C). (F) Growth curve of V. mali in (E). The half maximal effective concentration (EC50) for each fungus was calculated and is indicated in blue. (G,H) Alternaria solani and Colletotrichum higginsanum are not sensitive to decursin. For all these in vitro growth assays, 3 mm‐diameter mycelial discs were inoculated onto the medium with the indicated concentration of decursin. [file MPP-26-e70101-s006.pdf]

Supplementary Figure 4

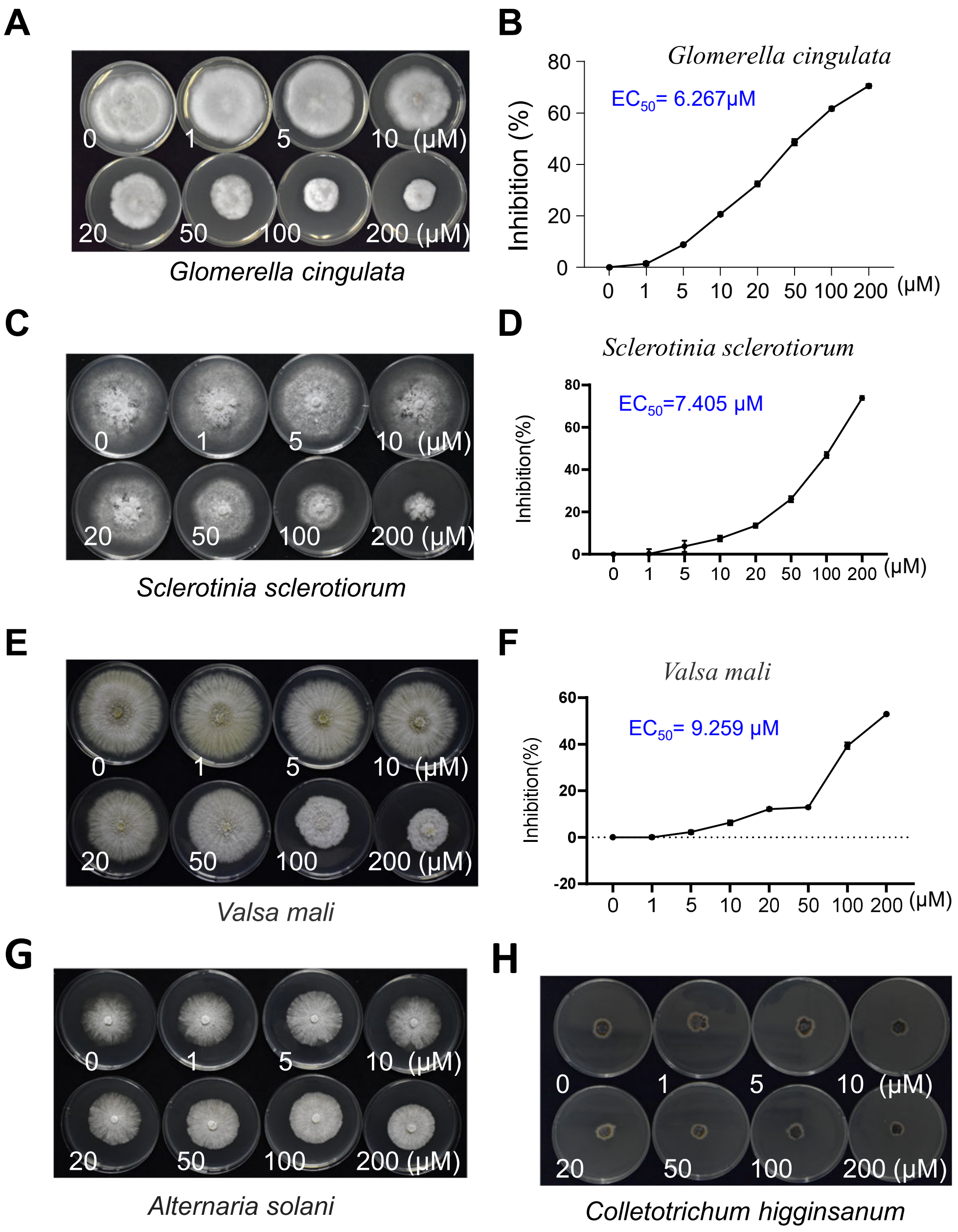

Supplementary Figure 4. The sensitivity of phytopathogens to decursin. Mycelial growth of *Glomerella cingulata* (A), *Sclerotinia sclerotiorum* isolate (C) and *Valsa mali* isolate (E) treated with different concentrations of decursin. (B) Growth curve of *G.cingulata* in (A). (D) Growth curve of *S.sclerotiorum* in (C). (F) Growth curve of *V.mali* in (E). The half maximal effective concentration (EC_{50}) for each fungus was calculated and is indicated in blue. (G-H) *Alternaria solani* and *Colletotrichum higginsanum* are not sensitive to decursin. For all these *in vitro* growth assays, 3 mM dimeter of the mycelial were inoculated onto the medium with the indicated concentration of decursin.
