# Supplementary material for: Decursin, Identified via High‐Throughput Chemical Screening, Enhances Plant Disease Resistance via Two Independent Mechanisms
Source: Mol Plant Pathol. 2025 Jun 1;26(6):e70101. doi: 10.1111/mpp.70101 (PMC12127108; doi:10.1111/mpp.70101)
Supplement: Supplementary file 5 — Figure S5. The growth of Pseudomonas syringae pv. tomato (Pst) DC3000 and Ralstonia solanacearum under decursin treatment. (A) Single colony of Pst DC3000 was inoculated on the media with different concentrations of decursin. (B) The growth curve of Pst DC3000 with indicated concentration of decursin. Data are mean ± SE (n = 3). (C) The growth of single colony of R. solanacearum treated with different concentrations of decursin. (D) The growth of R. solanacearum under the indicated concentration of decursin in a liquid medium. Data are mean ± SE (n = 3). [file MPP-26-e70101-s001.pdf]

Supplementary Figure 5

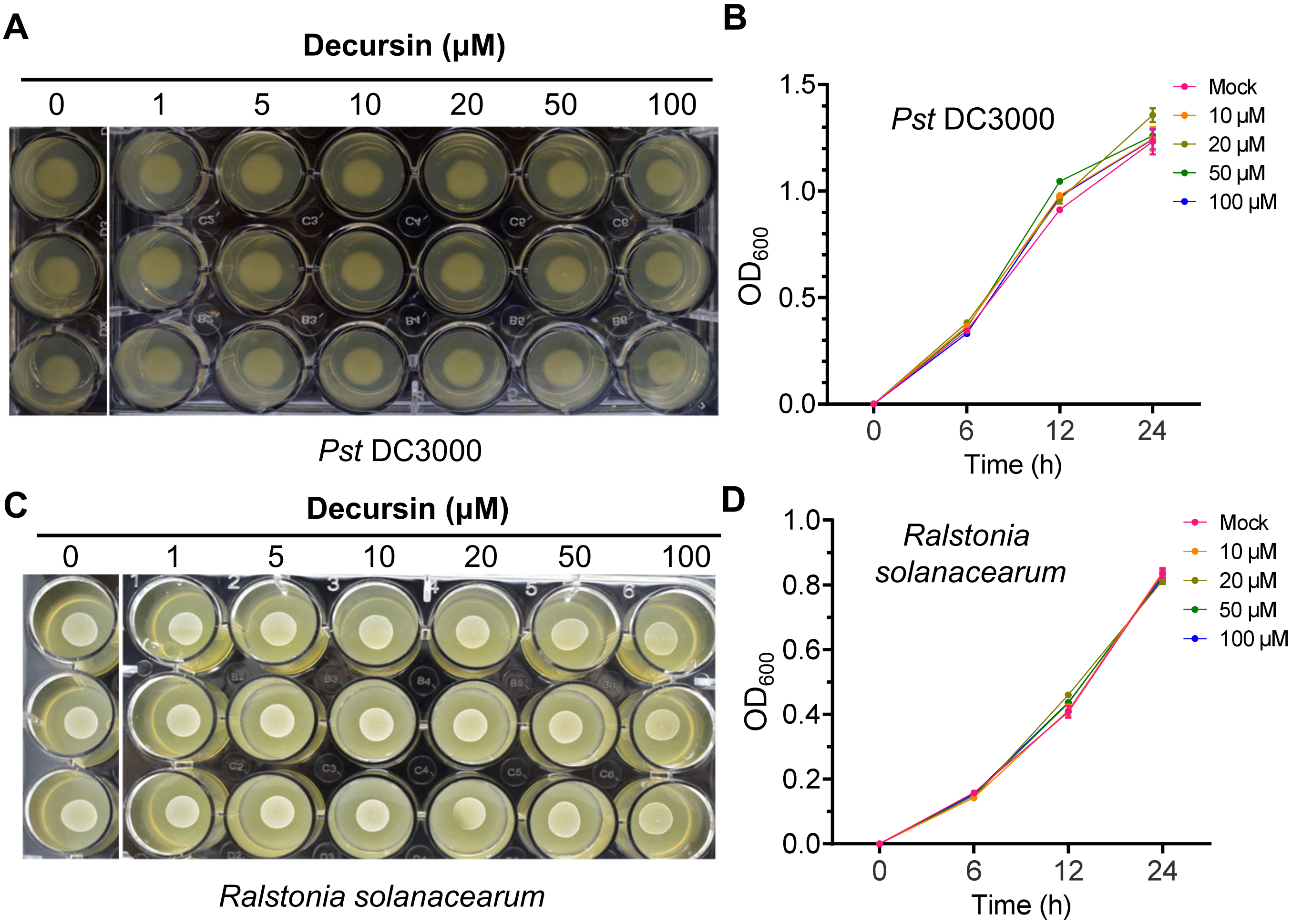

Supplementary Figure 5. The growth of *Pst* DC3000 and *Ralstonia solanacearum* under decursin treatment. (A) Single colony of *Pst* DC3000 was inoculated on the media with different concentrations of decursin. (B) The growth curve of *Pst* DC3000 with indicated concentration of decursin. Data are mean ± SE (n = 3). (C) The growth of single colony of *R. solanacearum* treated with different concentrations of decursin. (D) The growth of *R. solanacearum* under the indicated concentration of decursin in a liquid medium. Data are mean ± SE (n = 3).
