# Supplementary material for: Decursin, Identified via High‐Throughput Chemical Screening, Enhances Plant Disease Resistance via Two Independent Mechanisms
Source: Mol Plant Pathol. 2025 Jun 1;26(6):e70101. doi: 10.1111/mpp.70101 (PMC12127108; doi:10.1111/mpp.70101)
Supplement: Supplementary file 6 — Figure S6. The effects of decursin analogues on the growth of Botrytis cinerea and Fusarium graminearum in vitro. Fresh fungal mycelia with a diameter of 3 mm were inoculated onto the medium with the indicated concentration of the analogues of decursin. [file MPP-26-e70101-s004.pdf]

Supplementary Figure 6

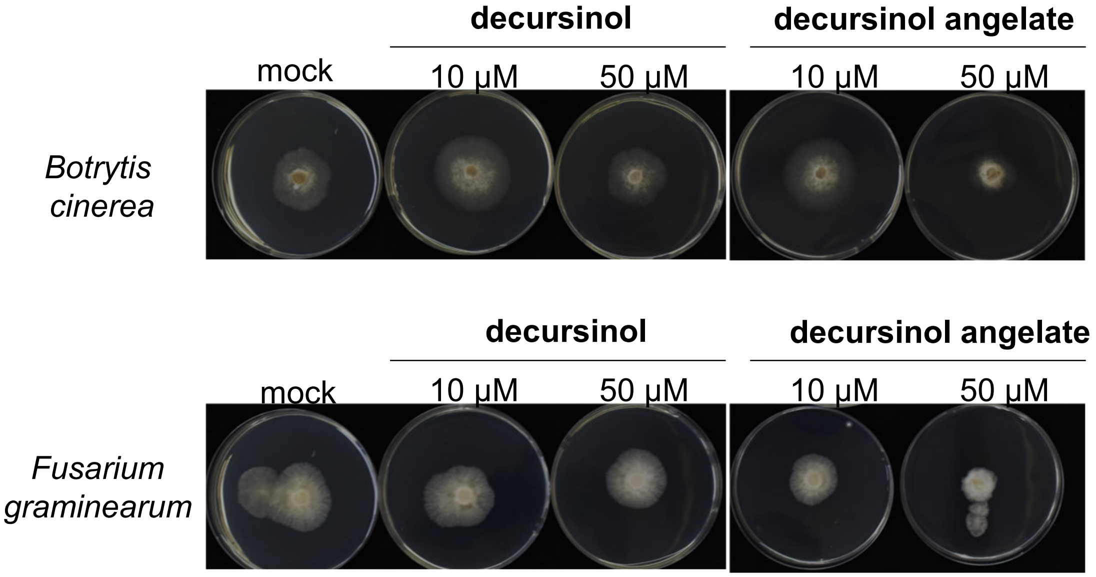

Supplementary Figure 6. The effects of decursin analogs on the growth of *B. cinerea* and *F. graminearum in vitro*. Fresh fungal mycelia with a diameter of 3 mM were inoculated onto the medium with the indicated concentration of the analogs of decursin.
