# Supplementary material for: Decursin, Identified via High‐Throughput Chemical Screening, Enhances Plant Disease Resistance via Two Independent Mechanisms
Source: Mol Plant Pathol. 2025 Jun 1;26(6):e70101. doi: 10.1111/mpp.70101 (PMC12127108; doi:10.1111/mpp.70101)
Supplement: Supplementary file 7 — Figure S7. The growth of phytopathogens treated with scopoletin. (A) Chemical structure of scopoletin. (B) The growth of Valsa mali, Sclerotinia sclerotiorum and Botrytis cinerea on the medium with 50 μM scopoletin. Discs of 3 mm diameter of fungal mycelia were inoculated on the medium. [file MPP-26-e70101-s007.pdf]

Supplementary Figure 7

A

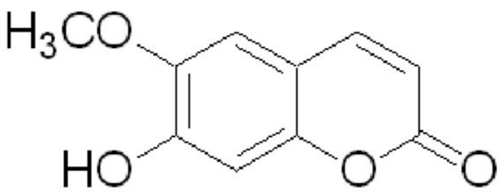

Scopoletin

B

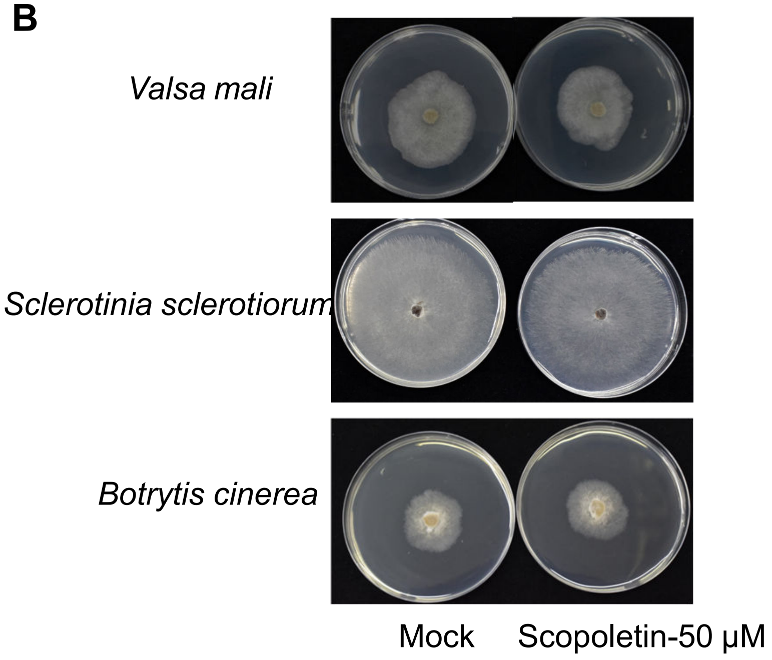

Supplementary Figure 7. The growth of phytopathogens treated with scopoletin. (A) Chemical structure of scopoletin. (B) The growth of *V. mali*, *S. sclerotiorum* and *B.cinerea* on the media with 50 μM scopoletin. 3 mM fungal mycelia were inoculated on the medium.
