# Supplementary material for: Decursin, Identified via High‐Throughput Chemical Screening, Enhances Plant Disease Resistance via Two Independent Mechanisms
Source: Mol Plant Pathol. 2025 Jun 1;26(6):e70101. doi: 10.1111/mpp.70101 (PMC12127108; doi:10.1111/mpp.70101)
Supplement: Supplementary file 8 — Table S1. List of primers for plasmid construction. [file MPP-26-e70101-s005.pdf]

Supplemental Table 1. List of primers for plasmid construction.

pFRK1-TOPO F	CACCATCCCTGACAGTGAACTTCATTG
pFRK1-TOPO R	TTACTTAATTGAGCTGCTTTCTCTG
