# Supplementary material for: Decursin, Identified via High‐Throughput Chemical Screening, Enhances Plant Disease Resistance via Two Independent Mechanisms
Source: Mol Plant Pathol. 2025 Jun 1;26(6):e70101. doi: 10.1111/mpp.70101 (PMC12127108; doi:10.1111/mpp.70101)
Supplement: Supplementary file 9 — Table S2. Primers used in the reverse transcription‐quantitative PCR. [file MPP-26-e70101-s009.pdf]

Supplemental Table 2. Primers used in the qRT-PCR.

AtPR1-qRT-PCR-F	TTCTTCCCTCGAAAGCTCAA
AtPR1-qRT-PCR-R	AAGGCCCACCAGAGTGTATG
AtPR2-qRT-PCR-F	GCTTAGCCTCACCACCAATG
AtPR2-qRT-PCR-R	CCCGTAGCATACTCCGATTT
AtFRK1-qRT-PCR-F	CGGTCAGATTTCAACAGTTGTC
AtFRK1-qRT-PCR-R	AATAGCAGGTTGGCCTGTAATC
AtWRKY29-qRT-PCR-F	ATCCAACGGATCAAGAGCTG
AtWRKY29-qRT-PCR-R	GCGTCCGACAACAGATTCT
AtWRKY30-qRT-PCR-F	AGCCAAATTTCCAAGAGGAT
AtWRKY30-qRT-PCR-R	GCAGCTTGAGAGCAAGAATG
AtWRKY33-qRT-PCR-F	GGTCACAACAATCCGGAAGA
AtWRKY33-qRT-PCR-R	GGAGAGACAAGAGAAGGAGAGA
AtWRKY53-qRT-PCR-F	TCACCGAGCGTACAACTTATTCC
AtWRKY53-qRT-PCR-R	CGTTTATCGATGCCGGAGATT
AtPAL1-qRT-PCR-F	GTGATTGGGTTATGGAGAGTATGA
AtPAL1-qRT-PCR-R	TGTTGAGGAAACTGGTAATTGCTT
AtPAL2-qRT-PCR-F	GAGCTTAAGGCTGTGCTTCC
AtPAL2-qRT-PCR-R	TTCGTTCCAAGCTCTTCCCT
AtNHL10-qRT-PCR-F	TTCCTGTCCGTAACCCAAAC
AtNHL10-qRT-PCR-R	GTCCTAGACTGTCCGGCGTT
ERF104qPCR-F	TAAACCGCCCTTACCGAATC
ERF104qPCR-R	TGTTCGGATCCCTAATCTCC
ZAT10qPCR-F	CTTCTCCGATTCCTCCTTTG
ZAT10qPCR-R	GTGGTGGAAATCGGATCTTG
EF1αqPCR-F	CAGGCTGATTGTGCTGTTCTTA
EF1αqPCR-R	GTTGTATCCGACCTTCTTCAGG
